# Chitosan-Based Multifunctional Biomaterials as Active Agents or Delivery Systems for Antibacterial Therapy

**DOI:** 10.3390/bioengineering11121278

**Published:** 2024-12-16

**Authors:** Meng Wang, Yue Wang, Geyun Chen, Hongyu Gao, Qiang Peng

**Affiliations:** State Key Laboratory of Oral Diseases & National Center for Stomatology & National Clinical Research Center for Oral Diseases, West China Hospital of Stomatology, Sichuan University, Chengdu 610041, China

**Keywords:** antimicrobial, drug delivery, bioactive materials, antibiotics, bacterium

## Abstract

Antibiotic therapy has been a common method for treating bacterial infections over the past century, but with the rise in bacterial resistance caused by antibiotic abuse, better control and more rational use of antibiotics have been increasingly demanded. At the same time, a journey to explore alternatives to antibiotic therapies has also been undertaken. Chitosan and its derivatives, materials with good biocompatibility, biodegradability, and excellent antibacterial properties, have garnered significant attention, and more and more studies on chitosan and its derivatives have been conducted in recent years. In this work, we aim to elucidate the biological properties of chitosan and its derivatives and to track their clinical applications, as well as to propose issues that need to be addressed and possible solutions to further their future development and application.

## 1. Introduction

Bacteria are an essential element of microorganisms in the human body, and most bacteria are commonly found in the gastrointestinal tract, oral cavity, skin, and other areas [1,2,3]. Although it is reported that some bacteria colonizing the human body are beneficial in human metabolism and immunity, e.g., probiotics, it is undeniable that bacterial infections have a profound impact on human health by affecting organs and tissues [1,3,4,5]. Controlling bacterial infections, lessening the health problems caused by them, and reducing deaths caused by these infections have become priorities for global public health organizations [6]. The diseases caused by bacterial infections are diverse, including infectious syndromes occurring at some common sites, e.g., respiratory tract infections, blood infections, urinary tract infections, etc. [5]. Recently increasing evidence has also shown that several cancers are highly associated with bacterial infections [7]. The introduction of antibiotics into clinical practice in 1940 was a great milestone in the control of bacterial infections, saving countless lives. However, at the same time, bacterial resistance has appeared in public populations as a result of their inappropriate use [8]. While antibiotic resistance is a predictable consequence of the use of antibiotics, abuse of antibiotics plays an essential role in the process of global bacterial resistance, which has been described as “one of the greatest global threats of the 21st century” [9,10]. The consequences arising from antibiotic resistance are immeasurable, which not only increase mortality and morbidity but at the same time incur significant costs in regards to medical resources and the social economy [11]. Therefore, developing new therapeutic approaches for reducing the usage of antibiotics is crucial, and functional biomaterials have shown their unpredictable potential in this area [12].

In recent years, studies of chitosan (CS) and its derivatives in the field of antimicrobials have attracted global attention [13,14]. Chitosan, derived from chitin, one of the most abundant natural polysaccharides in the world, has a variety of applications in biomedicine, but its remarkable antioxidant and antimicrobial activities have attracted significant attention. When employed as an antimicrobial agent, it can be used either alone or combined with other polymers. CS possesses far-ranging antibacterial activity, including action against Gram-negative bacteria, Gram-positive bacteria, and fungi, all of which are highly sensitive to CS [15]. As for the antibacterial properties of CS, according to previous studies, several antibacterial mechanisms have been put forward, and the most acceptable mechanism is that the destruction of cell structure is led by the interaction between positively charged chitosan molecules and negatively charged microbial cell membranes [15,16,17]. However, it is worth noting that the multiple antibacterial mechanisms of CS may occur at the same time. Its molecular weight (MW) and degree of acetylation (DA) also play a role in antibacterial activity. It is shown that the lower the MW and DA, the higher the free amino group present in CS and the more effectively CS can bind to the bacterial surface; thus, the antimicrobial activity can increase [18,19].

Attempts have been made to use CS and its derivatives as carriers to deliver drugs for antibacterial therapy, with satisfactory results, in addition to the use of their natural antimicrobial properties for antibacterial experiments [20]. Among the biodegradable polymers, CS is the only one exhibiting cationic properties; thus, its electrostatic interaction with anionic drugs makes it more advantageous compared to anionic materials for use as carriers. Moreover, it can facilitate the controlled release of anionic drugs [21]. Its cationic properties may also simultaneously benefit the mucoadhesive properties of CS because the mucus gel layer always exhibits an anionic substructure, distinguishing CS among other biodegradable polymers [21,22,23]. In addition, the excellent transfection-enhancing properties, permeation-enhancing properties, and efflux pump-inhibitory properties of CS make it an eminent drug delivery carrier. As for the binding of CS to drugs, the abundant primary amino groups of CS provide reaction sites for each reaction, allowing it to couple with small-molecule drugs [21,24]. Su et al. designed a nanocomposite system composed of CS, raspberry-like silver NPs, and graphene oxide; graphene oxide helps release silver NPs and GO for killing *E. coli* and *S. aureus*, showing higher antimicrobial properties of this multi-drug release system [21].

**Figure 1:**
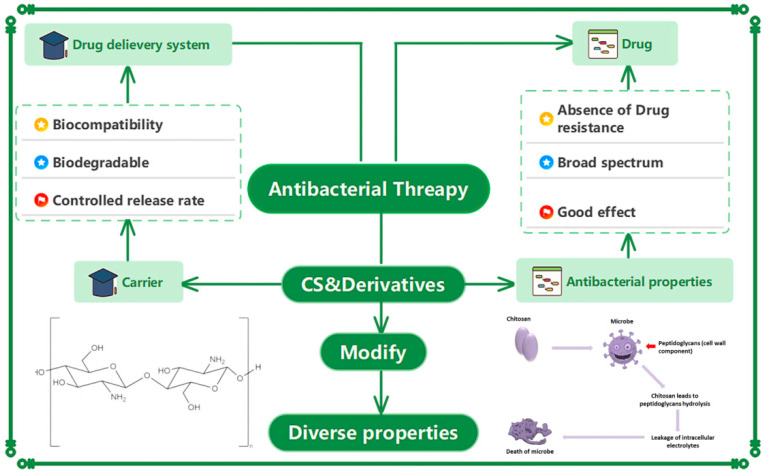
Applications of CS and its derivatives in antibacterial therapy.

This review aims to provide a comprehensive understanding of the antibacterial activity of multifunctional CS and its derivatives, as either direct antibacterial agents or drug delivery systems (Figure 1). Through the summary and discussion of previous studies, we elaborate the functions, explain the advantages and disadvantages, and discuss the future directions of CS-based multifunctional biomaterials in antibacterial therapy.

## 2. Antibacterial Mechanisms of Chitosan-Based Biomaterials

CS is famous for its strong natural antibacterial activity, but its application is limited due to its low solubility in water [25]. Therefore, a series of CS derivatives have been obtained by chemical modification, adjusting the physical, chemical, and biological characteristics of CS itself, broadening its applications. Numerous forms of CS derivatives can be used in a variety of situations due to their high performance [26]. CS, comprising functional groups such as hydroxyl and amino groups, can react with reagents to modify itself, thereby introducing hydrophilic groups such as carboxylic acid, sulfate, and phosphate groups, or introduce hydrophobic groups through the acylation of fatty acids and the alkylation of hydrocarbon chains to obtain a variety of CS derivatives [27,28,29]. CS and its various derivatives differ in their antimicrobial mechanisms.

### 2.1. Chitosan

**Figure 2:**
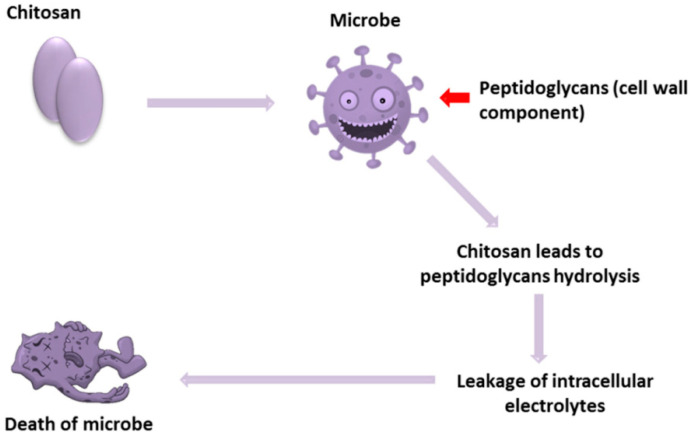
Possible mechanisms of antibacterial activity of CS. Reprinted from Ref. [42].

Several hypotheses about the mechanism of CS working against microorganisms are put forward, and the most widely acknowledged is based on the cationic properties of CS. As Figure 2 shows, the positively charged amine groups (NH_3_^+^) are revealed to interact with the negatively charged bacterial cell membrane, neutralizing and reversing the surface charge of the bacteria, disrupting the cell integrity. Therefore, the number of amino groups has an important impact on the antimicrobial ability of CS [15,30,31]. In addition, there are various other possible mechanisms. For example, CS can penetrate into the nucleus of the microorganism and then bind to its DNA, thereby inhibiting the microbial mRNA and preventing the synthesis of protein [32,33,34]. Additionally, low MW (≤50 kDa) CS and its nanoparticles can enter bacteria to inhibit the expression of DNA by combining with it [35]. Since CS is generally thought to damage the outer membrane to a greater extent, rarely penetrating the cell, this mechanisms may seldom occur [36]. The third mechanism suggests that CS has excellent metal binding ability, which causes the amino group of CS to selectively bind to essential metals. For example, the amino group can bind to divalent ions such as Ca^2+^ in the bacterial cell wall [35], then to chelate metals in the extracellular area, blocking the flow of bacteria-required nutrients to inhibit microbial growth. At the same time, it will activate the defense responses in the host. But the interaction sites of CS and metal are limited; therefore, it is not considered as the decisive mechanism [37,38,39,40]. Another suggestion is that CS can form a blocking layer outside the bacterial cell, causing its death due to lack of nutrients and oxygen. The intensity of the antibacterial action of CS is related to its molecular size. CS and its derivatives with high MW cannot enter directly into the cells but interact with the substances of the cell surface. However, low MW CS and its derivatives can enter the cells more easily, binding to DNA while inhibiting mRNA’s binding to proteins to disrupt the process of DNA transcription [41].

### 2.2. Quaternized Chitosan

Quaternized chitosan is a type of CS derivative prepared by grafting quaternary ammonium units on CS. Such modification improves the performance of this derivative by increasing its water solubility, optimizing its antibacterial and antioxidant properties, which has a broad impact on its potentially applications [25].

The most significant advantage of quaternized chitosan as an antimicrobial agent is its cationic configuration. Quaternized chitosan has higher positive charge, thanks to the ammonium group in the structure, making the electrostatic interaction between it and bacteria containing anions, which is the main mechanism of antibacterial function, stronger. Then, the integrity and permeability of the cell membrane will be affected, resulting in content leakage. Moreover, the hydrophobic alkyl group on quaternized chitosan can also interact with the inner surface of the bacterial cell wall to stimulate bacterial death [25,43]. And the smaller the alkyl group, the easier it binds to the cell wall and the stronger the antimicrobial activity it shows. For example, the antibacterial property of N,N,N-trimethyl chitosan (TMC) is stronger than that of N,N-diethyl-N-methyl-quaternary ammonium chitosan (DMCHT) because the methyl group is smaller than the ethyl group [44]. Moreover, compounding with the membrane is the unique antimicrobial mechanism for TMC to achieve a potent antibacterial effect. It is also reported by further study that the higher the positive charge possessed by TMC, the greater its performance, while it will decrease under acidic conditions along with the decreasing pH [45,46,47,48].

### 2.3. Acylated Chitosan

**Figure 3:**
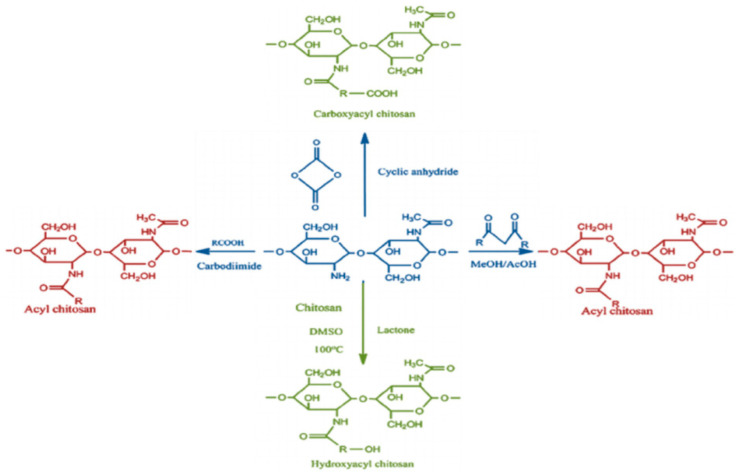
N-acylation of chitosan. Reprinted from Ref. [42].

Acylation is the most common modification of CS, which introduces aliphatic or aromatic acyl groups into the CS molecules through the acylation reaction, including N-acylation and O-acylation (Figure 3). N-acylated chitosan is often used as a carrier in drug delivery systems or in biological scaffolds, while O-acylated chitosan is more often used to enhance the hydrophobicity and stability of materials [33,42,49,50].

**Figure 4:**
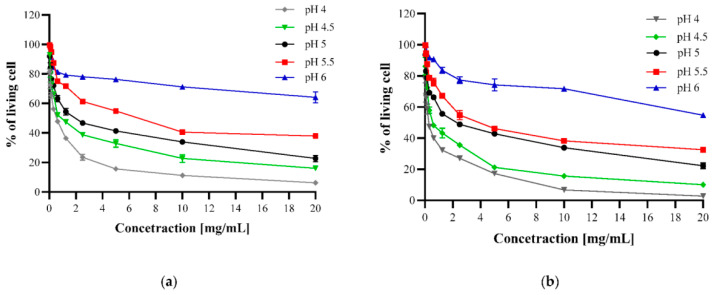
Antibacterial properties of CS (**a**) or CH_LA (**b**) against *E. coli* at different pH levels. Reprinted from Ref. [51].

It is reported that N,O-acylated chitosan such as CH_LA displays more efficient antibacterial activity than CS in a suitable pH range (*E. coli* in the pH range of 4–4.5) (Figure 4). This indicates that the improvement in the antibacterial properties of CS may be related to the fatty acids in the solution or the change in macromolecular behavior. Moreover, it was found that the hydrophobic interaction between CH_LA and the bacterial cell constituents seems to contribute to a stronger bond [51].

### 2.4. Carboxylated Chitosan

Figure 3 shows that the carboxylation reaction primarily uses glyoxylic acid or chloroalkane acid to react with CS and form -COOH groups, which exhibit better water solubility. Compared with single CS, carboxylated chitosan displays better thickening, heat preservation, and film formation properties [33].

The use of carboxymethylation reactions is a simple way to endow CS with new properties [33]. It has been shown that carboxymethyl chitosan (CMC) performs better than CS in regards to its antimicrobial property. In addition to the basic mechanism through which its cationic structure will bind with the microorganisms’ anionic structure, CMC displays another way to strengthen its antimicrobial abilities through the introduction of a carboxymethyl group into the CS molecular chain, giving the molecule hydrophilic ends, and these can form weak interactions with CS, thereby enhancing its antibacterial activity [52]. Among different carboxymethyl chitosans, O-carboxymethyl chitosan (OCM) still contains -NH_2_ groups, since the substitution occurs only in the -OH groups, and thus, it shows stronger antibacterial activity than does CS. In contrast, N,O-carboxymethyl chitosan (NOCM) shows compromised antibacterial activity since -NH_2_ groups are also carboxylated [35].

### 2.5. Chitosan–Metal Complexes

Studies have revealed that adding metals to CS can significantly enhance its antibacterial activity; for example, the chelation of Zn^2+^ and Ag^+^ with CS can significantly enhance its antibacterial activity, and the loading of Cu^2+^ and Mn^2+^ increase the antibacterial activity of CS when compared to that of CS alone. As mentioned above, this is closely related to the antibacterial mechanism of CS itself, and the interaction sites of CS and metal ions are pH-dependent. The higher the pH, the greater the ability of the amino groups in the CS molecule to absorb metal cations by chelation [31].

Currently, metal nanoparticles and metal oxide nanoparticles (such as zinc oxide nanoparticles) are increasingly incorporated into CS films to enhance their antibacterial properties and to fit them for biomedical applications [53]. They not only have significant advantages in improving the antibacterial properties of CS films, but they can also stimulate tissue growth and healing and are used in drug delivery systems as carriers of various bioactive agents, playing a significant role in impacting antibacterial ability of the body in a variety of ways [54].

### 2.6. Chitosan-Based Hybrid System

In recent years, the development of hybrid composites has been greatly promoted, with a wide range of applications regarding antibacterial properties. For CS, hybrid composites can be combined in an organic–organic manner with alginate, proteins, etc., or with inorganics, such as TiO_2_, etc. in an organic–inorganic manner to prepare nanoparticles, thus effectively improving the performance of each component [55].

CS–TiO_2_ in inorganic hybrid composite materials is regarded as a promising material, demonstrating excellent antibacterial activity against bacteria, yeasts, and molds. TiO_2_ has been found to be present on the surface of this hybrid composite material, thereby generating electron-hole pairs without the requirement of light-induced radiation, thus enhancing its antibacterial activity. Li and Xiao et al. also found that bacterial inactivation was associated with electrostatic forces between the cell membrane and the composite and the oxidative stress caused by reactive free radicals generated by light [55]. Organic hybrid composite nanoparticles also show great promise in regards to antibacterial activity; notably, CSNPs can stimulate the production of ROS in bacterial cells and destroy cellular structures and components, further enhancing their antibacterial properties. CSNPs also exhibit the advantages of interfering with bacterial biofilm formation, promoting biofilm eradication, and penetrating biofilm to directly destroy bacterial cells.

### 2.7. More Chitosan Derivatives

Due to the modifiability of CS, numerous groups with distinct properties have been grafted into CS in through research endeavors, endowing it with greater potential. For instance, combining thiol-functional coupling reagents with the primary amino groups of CS to obtain thiolated CS strengthens its own properties (providing enhanced mucosal adhesion, enhanced transmembrane transport permeability, good biodegradability, and the function of inhibiting enzyme degradation) through this modification approach; modified sulfated CS exhibits biological activities such as antibacterial, antiviral, antioxidant, and enzyme inhibition; thiourea CS is obtained through ammonium thiocyanate heated with CS to introduce thiourea groups, which can chelate with silver ions and significantly enhance the antibacterial activity of CS. Additionally, there are many CSs modified for antibacterial and therapeutic purposes that are currently under investigation [56].

## 3. Chitosan-Based Biomaterials as Direct Antibacterial Agents

**Figure 5:**
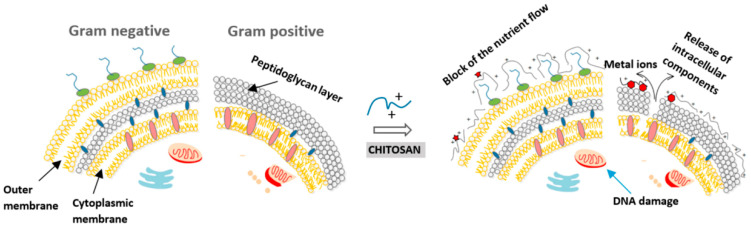
Hypothesis of antibacterial activity towards Gram-positive and Gram-negative bacteria. Reprinted from Ref. [35].

Differences regarding their antimicrobial properties exist not only between CS and its derivatives, but also in the reactions with different types of microorganisms for the same CS. Differences in the structure between Gram-positive and Gram-negative bacteria cells leads to their different responses to CS and its derivatives. The cell wall of Gram-positive bacteria includes peptidoglycan and teichoic acid, ensuring the structural stability of the cell wall [15], and Gram-negative bacteria has a thick and highly negatively charged peptidoglycan layer. The abundant lipopolysaccharide in the outer membrane also provides the Gram-negative bacteria with hydrophilic surface properties (Figure 5) [57,58]. Therefore, for different types of bacteria, understanding the different natural antibacterial activities of CS and its derivatives helps in the selection of suitable materials when it comes to antibacterial applications.

### 3.1. E. coli

*Escherichia coli* (*E. coli*) is a rod-shaped Gram-negative bacterium which commonly colonizes in the gastrointestinal tract of endothermy animals, which has a greatly increased the exposure to antibiotics, making it easier to develop resistance to the antibiotics used by the host [59,60]. In addition, *E. coli* can easily form biofilms to escape the host immune response, while the periodic release of biofilms can cause persistent infections [61,62].

**Figure 6:**
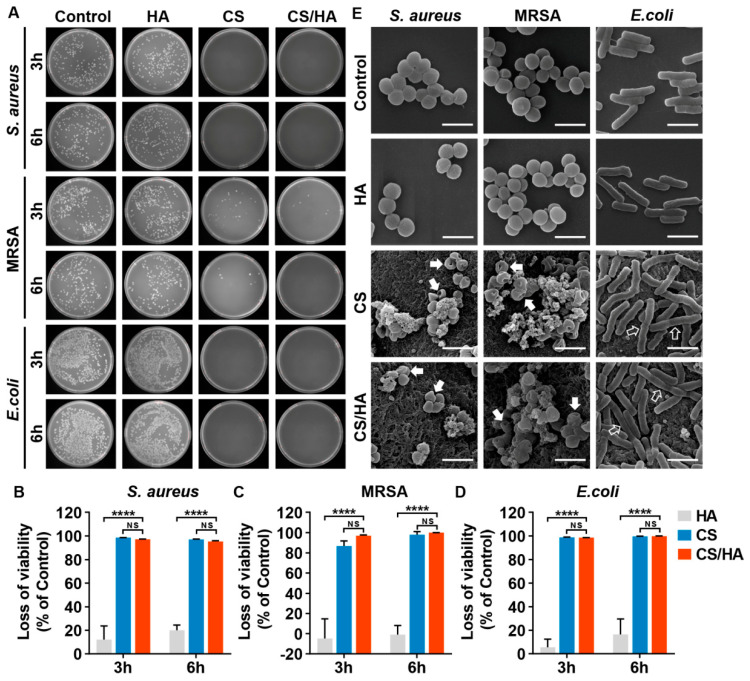
In vitro antibacterial activity of HA, CS, and CS/HA hydrogels against *S. aureus,* MRSA and *E. coli*. (**A**) Bacterial colonies grown on agar plates after treatment for 3 and 6 h. (**B**–**D**) Bacterial viability loss after treatment. (**E**) Bacteria morphology examined by SEM after treatment for 6 h (white solid arrows indicate the holes on bacterial cells, and white hollow arrows indicate the elongated bacterial cells). Scale bar: 2 μm. All data are presented as mean ± SD (n = 3). Statistical analysis: NS, non-significant; **** *p* < 0.0001. Reprinted from Ref. [64].

It has been shown that in addition to the CS-based antibacterial effects such as the destruction of bacterial cell membrane structures, compared with single CS, CS derivatives, with a variety of modified groups introduced into CS, can enhance its antibacterial properties against *E. coli* and inhibit its growth. The general mechanisms of the derivatives’ antimicrobial ability include the prevention of *E. coli* from adhesion and the reduction of biofilm formation, thus restraining its metabolic activity. The oleoyl-chitosan nanoparticles prepared by Xing et al. and the sulfonated chitosan synthesized by Huang et al. both showed satisfying antibacterial effects against *E. coli* [63]. Some studies have even found that TMC derivatives can achieve a bactericidal effect on *E. coli* [42]. Lin et al. evaluated the CS, HA, and CS/HA hydrogels for e. coli antibacterial properties. As shown in Figure 6, almost no colonies were identified on the AGAR plate of the CS group, which reveals a high viability loss for pathogens (>95%, Figure 6B–D), and through scanning electron microscopy, it was determined that elongation in the morphology has taken place in *E. coli*, showing the effect of CS on the antibacterial activity against *E. coli* [64].

Wang et al. prepared sulfonated chitosan, including ISCS (N-isonicotinic sulfonate CS), HSCS (N-(sodium 2-hydroxypropylsulfonate) CS), and SCS (N-sulfonate CS), by direct sulfonation and introduced chitosan with compounds containing sulfonic acid groups, which showed good antibacterial activity against *E. coli* and *S. aureus* [65]. In detail, the antibacterial rate of 2.5 mg/mL SCS against *E. coli* was 63.7%, and the antibacterial rate of ISCS against *E. coli* was 79.2% (the highest among all the derivatives). The cell membrane damage was examined by measuring the protein concentrations in the suspension. As a result, the protein content in the ISCS group was the highest, indicating that ISCS caused the most severe damage to the bacterial membrane [65]. In another work, Wang et al. synthesized the chitosan derivatives containing six-membered heterocyclics and showed that the antibacterial and antibiofilm activity of CS could be enhanced by increasing the hydrophobicity, alkalinity, and charge density of the substitution groups [66].

### 3.2. S. aureus

As a well-known bacterial pathogen, *Staphylococcus aureus* (*S. aureus*), a Gram-positive bacterium, has a close connection with many diseases occurring in humans. Drug resistance in *S. aureus* is very common, and one of the most famous examples is *Methicillin-resistant Staphylococcus aureus* (*MRSA*) [10,67]. MRSA is a common pathogen that can cause a variety of refractory infectious diseases. Its high drug resistance greatly weakens the effects of traditional antibiotic treatment [12,68,69,70]. Releasing β-lactamases is the main mechanism by which it develops drug resistance, which inactivates penicillin and its derivatives [71]. In addition, *S. aureus* can also form a biofilm [63].

CS and its derivatives have been found in previous experiments to exert inhibitory effects on the planktonic and sessile growth of *S. aureus* [63,72,73,74]. However, when it comes to whether CS can limit the biofilm-forming activities of *S. aureus*, the results of such experiments are polarized. Some insist that CS cannot inhibit the formation of biofilm, while others believe that CS is a potent inhibitor for *S. aureus* biofilm growth [75,76,77,78]. Wang et al. demonstrated that the sulfonated chitosan SCS and ISCS (2.5 mg/mL) showed a 100% antibacterial rate against planktonic *S. aureus*, and ISCS showed a greater ability to remove the *S. aureus* biofilm [65]. The six-membered heterocyclic chitosan derivatives showed a 100% antibacterial efficiency against planktonic *S. aureus*, and more than 80% biofilm removal rates [66].

### 3.3. P. aeruginosa

*Pseudomonas aeruginosa* (*P. aeruginosa*) is a Gram-negative bacterium with an etiology of nosocomial infection. Considered to be one of the most life-threatening bacteria, it easily causes chronic infection in individuals with compromised immune systems [79]. *P. aeruginosa*’s outstanding fitness, inherent resistance to antibiotics, and capacity for biofilm formation pose challenges for its treatment. Its facile acquisition of antibiotic resistance results in the emergence of multidrug-resistant or even pandrug-resistant strains [79,80].

In *P. aeruginosa*, the formation of virulence factors and biofilm are regulated by quorum-sensing (QS), an intercellular communication system adjusting the expression of genes involved in virulence factors and biofilm [81,82]. Recent evidence suggests that CS may interfere with QS in *P. aeruginosa* [83,84]. Piras et al. found that N-trimethyl chitosan derivatives have showed excellent antibacterial, anti-biofilm, and anti-adhesion properties against *P. aeruginosa* due to their low MW and high degree of substitution, proposing that they can be used as therapeutic agents for bacterial infections [85].

**Figure 7:**
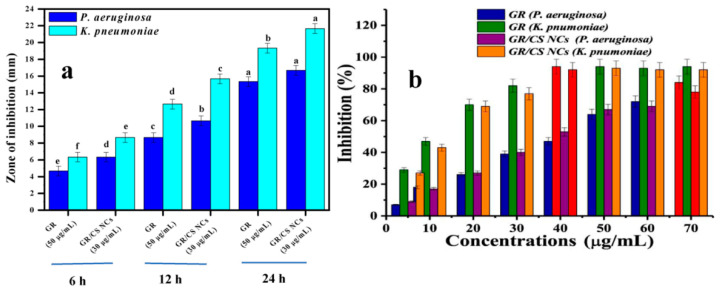
Antimicrobial activity (**a**) and biofilm inhibition ability (**b**) of GR and GR/CS NCs against *P. aeruginosa* and *K. pneumoniae*. Reprinted from Ref. [86].

A recent study shows the dose- and time-dependent antibacterial activity of graphene/chitosan nanocomposites (GR/CS NCs) against *P. aeruginosa* (Figure 7). Upon treatment with GR/CS, many morphology changes in *P. aeruginosa* cells, including surface irregularity, membrane collapse, bubble formation, and cell surface leakage, were observed [86]. In their study of chitosan alkylation, 11-carbon and 3-carbon alkyl chains were grafted onto CS. The 11-carbon alkyl chains then spread throughout the biofilm of *P. aeruginosa*, destroying the biofilm biomass and reducing living cells in the biofilm [87].

### 3.4. S. mutans

*S. mutans* is the primary microorganism responsible for human dental caries. It rapidly adheres to the surface of the teeth in the oral cavity and secretes acid from sucrose, synthesizing extracellular polysaccharides from sugar to promote biofilm formation. This facilitates the permanent colonization of *S. mutans* on tooth surfaces and the local development of extracellular polymeric substances (EPS), providing a protective environment for acid-producing bacteria. This process promotes the gradual demineralization of dental hard tissue and is closely associated with the onset and progression of dental caries [88,89,90,91]. EPS plays a crucial role in promoting bacterial adhesion, cohesion, and resistance to environmental stress. It also hinders the diffusion of nutrients and metabolic products, thereby significantly impacting the cariogenic potential and bacterial virulence within biofilms [92]. Therefore, while dental caries is a multifactorial disease involving various microorganisms, the targeted inhibition of *S. mutans* is considered a viable approach for preventing dental caries.

Based on the biological functions of CS, question about whether CS can coordinate the prevention and treatment of dental caries has attracted great interest. The excellent antibacterial properties of CS make it a potential antibacterial biomaterial for coating implant frameworks, and CS/polyethylene glycol hydrogels and CS nanocomposites have been found to have great potential for dental caries treatment [90,93]. It is reported that after 6 h of exposure to a 1 mg CS implant (1% (*w*/*v*)), the viability loss of *S. mutans* exceeds 97%, and the CS film exhibits antibacterial effects against lower concentrations of *S. mutans* [15,93]. Furthermore, research has also found that CS combined with amelogenin-derived peptide QP5 hydrogel shows good resistance to *S. mutans* [93]. Wang et al. studied the effect of CS on *S. mutans* biofilm formation by preparing functional brackets coated with non-crosslinked bioactive CS (Br-CS), and the results showed that the CFU count of the Br-CS group incubated with *S. mutans* began to significantly decrease from 6 h, *S. mutans* was almost killed, and the formation of biofilm was completely inhibited, indicating that the CS coating exhibits an excellent inhibitory function, as well as antibacterial effects against *S. mutans* biofilm [90]. Utilizing citric acid as a multivalent anion, submicron chitosan particles exhibiting high dispersibility in aqueous solutions were synthesized via the phase separation method. Compared to CS alone, these particles demonstrated superior antibacterial activity against *S*. *mutans*, independent of lysozyme presence, which is attributable to their larger specific surface area. This increased surface area facilitates more interaction points between the free amino groups and the negatively charged cell wall of *S*. *mutans*. Furthermore, DCPs-1.0 exhibited greater inhibition of the insoluble glucan adhesion produced by *S*. *mutans* than did CS [94].

### 3.5. Fungus

The prevalence of fungal infections has been increasing over the last three decades, resulting in tens of thousands of deaths, but they have been largely neglected compared to diseases caused by other types of pathogenic microorganisms. In addition to antibacterial activity, CS also exhibits antifungal activity [95]. From the beginning of the study of plant pathogenic fungi to the study of a wider range of pathogenic fungi, the understanding of the antifungal properties of CS is gradually deepening. When it comes to the antifungal mechanism, research suggests that CS can react with the phospholipid of the fungal cell wall, causing damage of the fungal cell membrane, thus lead to the death of the fungus [42]. However, the contents of chitin and unsaturated fatty acids in the cell wall can also influence the tolerance of fungi to CS, i.e., fungi with less chitin and more unsaturated fatty acids in the cell wall are more sensitive to CS. Additionally, the physicochemical properties of CS, the degree of deacetylation, sample dispersion, pH value, etc., all have an impact on the antifungal activity of CS.

In addition, some derivatives of CS exhibit more potent antifungal activity than does CS, and even display fungicidal effects, which is determined by the groups grafted to CS, e.g., quaternary CS derivatives can show potent antifungal activity [42]. The modification of CS nanoparticles is also a way to enhance antifungal activity, which may be due to the higher surface charge density, larger surface area, and better cellular uptake function of the CS nanoparticles [96].

### 3.6. Multidrug-Resistant Strains (MDR)

The increasing use of antibiotics in the medical field over the last few decades has significantly increased the number of pathogens that are resistant to specific antibiotics, leading to the emergence of MDR [97]. What makes MDR a headache is its biofilm, which allows it to tolerate antibiotics and survive in harsh environments. MDR also regulates cell behavior through QS. Therefore, the study of antimicrobial therapy targeting MDR biofilms is of great significance for the eradication of MDR strains [98].

The positive charge of CS endows it with antibacterial properties. And recently, people have begun to prefer to use chitosan-based hybrid composite nanomaterials for MDR biofilm treatment. In addition to the antibacterial mechanism of CSNPs themselves (ROS effect, chelation, electrostatic interaction, etc.) and their stable anti-biofilm formation ability, a variety of hybrid composite materials endow them with functionally diverse antibacterial effects. Experimental cases including CS/AgNPs showed changes in the permeability of cell membrane by their adhesion to bacteria, Zn^2+^ in CS/ZnONPs attacked negatively charged bacteria, leading to leakage of the cell wall, and CuNPs-MAG/CS induced antibacterial effects through the ion exchange between magnesium and Cu ions, etc. [98].

### 3.7. Differences in Antibacterial Properties Among CS and Its Derivatives

The role of CS and its derivatives in antibacterial activities is significant. In addition to the widely accepted antibacterial theory, e.g., amino group binding to the cell membrane, CS and its derivatives also exhibit different antibacterial mechanisms against different bacteria.

In sulfonated chitosan, ISCS exhibited excellent antibacterial and anti-biofilm activities compared with those of HSCS and ISCS. This enhancement can be attributed to the introduction of pyridine salt structures into sulfonate chitosan, resulting in an increased positive charge and improved affinity with bacterial cell membranes, enhancing the antibacterial properties and anti-biofilm activity of CS [65]. The chemical modification of CS by introducing a six-membered hybrid ring showed that the presence of hydrophobic substituents, the increase in the alkalinity of the substituents, and the higher positive charge density all had a positive effect on the antibacterial activity of chitosan derivatives, while the addition of steric substituents reduced their antibacterial and anti-biofilm activities because the large substituents were not beneficial to the contact between CS and bacteria [66]. In the GR/CS assay, the antibacterial effect was not only derived from CS, but GR could disrupt 90% of biofilm formation in both Gram-positive and Gram-negative bacteria, and GR could penetrate into the polymer matrix of the biofilms, disrupting its three-dimensional structure, thereby inducing cell detachment and interfering with microbial adhesion [86]. The alkylated chitosan showed more significant interactions with the membrane phospholipids under the action of carvacrol, a better penetration of bacterial biofilm, and an anti-dynamic effect [87].

## 4. Chitosan-Based Biomaterials as Delivery Systems

**Figure 8:**
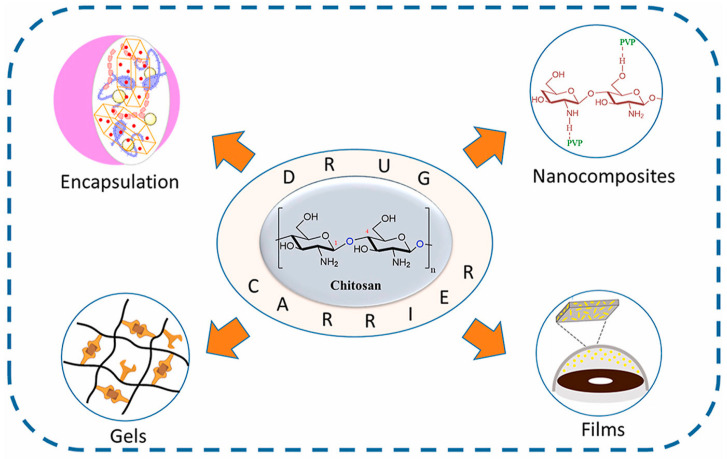
Diverse ways for CS to be used as a carrier. Reprinted from Ref. [100].

Chitosan is also a potential carrier for drug delivery [99]. According to Figure 8, due to the good biocompatibility and mucosal adhesion it possesses, its use as a carrier has also been widely researched. It is worth mentioning that drugs can be embedded in CS in a variety of ways [100]. Since the low solubility of CS has restrained its use as a carrier, obtaining chitosan derivatives by modification and functionalization is an important way to improve its performance. For example, ethylene glycol-chitosan-based (CNPs) carriers currently stand out as drug delivery carriers due to their excellent water solubility and biocompatibility [28,29,35].

In fact, chitosan drug delivery systems can be delivered by oral, ocular, nasal, pulmonary, periodontal, vaginal, skin, and transdermal routes [29,35,49,100], among which oral administration is the simplest, most effective, and safest method. At the same time, the emergence of nanotechnology has made the delivery vector options of oral drugs more diverse and has shown great advantages [100]. However, it is notable that the excellent mucosal adhesion properties and permeability properties of CS make it easier to isolate the drug in a local area, which allows the drug to be absorbed at a specific target, so CS is mainly used for local infections.

### 4.1. Delivery of Antibiotics

In recent years, the increasing morbidity and mortality caused by extensively drug-resistant bacteria have had a significant impact on global public health, resulting in noticeable social and economic threats. Furthermore, the cytotoxicity of antibiotics to the human body also adds a hidden danger to their use [100]. Systemic toxicity may result from systemic administration of some antibiotics because of insufficient cell penetration, and more seriously, kidney and liver diseases can occur due to such treatment [101].

Biocompatibility and a lack of cytotoxic effects are essential traits for an ideal drug carrier [100]. CS and its derivatives display obvious advantages in this regard. With the broad-spectrum antimicrobial properties of CS and its derivatives, they can be used as antibacterial agents to achieve a synergistic effect with the delivered antibiotics so as to enhance their antibacterial properties [102,103]. The synergistic effects between the CS-based carriers and the loaded antibiotics may play significant roles in enhancing the efficacy of antibacterial therapy. As a carrier, CS can provide a sustained release of antibiotics, allowing those drugs to exert long-lasting antibacterial activity. CS can also enhance the intracellular delivery of antibiotics via increasing the membrane permeation of the bacterial cells. Also, CS-based nanoparticles may break the integrity of biofilm and enhance the antibiofilm capacity of the loaded antibiotics. Natallia et al. increased local bioavailability and reduced systemic toxicity of the drug by coupling the peptide antibiotic colistin (CT) to succinylated chitosan [104]. It is worth mentioning that chitosan-based nanoparticles have shown great potential in regards to their antibacterial properties, and were evaluated for their incorporation into drug delivery systems. The outcomes of the studies lean toward a positive consensus. The smaller particle size endows chitosan nanoparticles with a larger surface area-to-volume ratio, which also endows it with a stronger drug-loading function and stronger antibacterial properties [105]. When loaded with antibiotics, CSNPs can effectively remove the biofilm and allow the antibiotics to act more effectively. Thus, the two promote each other synergistically, producing a stronger antibacterial effect. Fan et al. combined cephalosporin antibiotics and β-lactamase inhibitors with chitosan nanoparticle encapsulation, showing improved antimicrobial activity and lower cytotoxicity [101].

### 4.2. Delivery of Photosensitizers

As a new rising cancer treatment method, photodynamic therapy (PDT) has been widely accepted because of its lower injury rates, fewer side effects, and more effective avoidance of the potential drug resistance of chemotherapy. At the same time, photodynamic therapy has been gradually applied to antibacterial therapy as an adjuvant therapy, combining a non-toxic photosensitizer and light irradiation, producing ROS for sterilization, effectively eliminating the microbial membrane of the bacteria [93,106]. However, with their high hydrophobicity and nonspecific cytotoxicity, the clinical scenarios for photoexcited photosensitizers (PSs) are limited [107,108]. Therefore, finding a type of carrier with hydrophilicity and biocompatibility carriers for modifying PSs has become a priority for solving this problem [108].

CS and its derivatives stand out in this regard, especially the modified chitosan derivatives, which possess a higher water solubility. The modification of PSs with these can obviously improve the hydrophilicity, biocompatibility, and therapeutic effect of PSs [109,110,111]. Ding et al. successfully reduced the inherent nonspecific toxicity of the photosensitizer chlorin e6 by encapsulating it into ethylene glycol-chitosan nanoparticles (CNPs) [112]. At the same time, the application of CS in antibacterial photodynamic therapy has gradually become the focus of attention. Adding CS to the photosensitizer can improve the biological activity of antibacterial photodynamic therapy. It is reported that the chitosan–photosensitizer combination has the highest cellular uptake level and ROS production, and its antibacterial effect on bacteria is significantly increased [93,113,114].

### 4.3. Delivery of Antigen

Immunoreaction is an important mechanism for the body to reject pathogens and prevent bacterial infection. Vaccination is one of the leading ways to limit the spread of infectious diseases through immune response [33]. Compared with common intramuscular vaccines, more and more studies on mucosal vaccines have been conducted recently. It has been determined that mucosal immunity, as the immune system’s first-line defense, can defend against the invasion of pathogens at an early stage, with a faster onset, higher level, and longer duration than those noted for serum antibodies [115,116].

CS and its derivatives can open up the tight junctions between epithelial cells by altering the distribution of F-actin filaments [117], thus facilitating the transmembrane delivery of the drug. And compared with single chitosan nanoparticles, chitosan derivative nanoparticles exhibit better mucosal adhesion and water solubility [33]. Their mucosal adhesion property comes from their cationic traits, as they display an electrostatic interaction with negatively charged cell surfaces and mucus. And vivo mucin contains plenty of negatively charged sialic acid at physiological pH, so that they can bind to CS and its derivatives. Moreover, the longer the CS and its derivatives adhere to the mucous membrane, the longer the antigen particles carried as carriers remain, allowing them to bind to the cells and release the antigen with a higher probability [118]. Therefore, CS and its derivatives have become popular antigen-delivery carriers for antibacterial and infectious disease treatment. Quaternized chitosan and trimethyl chitosan and its various modified derivatives have been found to produce remarkable results as antigen vaccine carriers [119,120,121,122,123].

### 4.4. Delivery of Antibacterial Peptide

Antimicrobial resistance caused by the abuse of antibiotics is a global problem, but the emergence of antimicrobial peptides (AMPs) may be the solution. AMP is a protein molecule with a broad antibacterial spectrum which can avoid microbial resistance. More importantly, AMP shows potent antibacterial activity towards many drug-resistant bacteria [124,125], while the shortcomings of the clinical application of AMPs include its instability and cytotoxicity in the biological environment, making this an urgent problem to solve before generalizing its further research [126].

Chitosan nanoparticles (CS-NPs) appear to be an effective delivery carrier for AMPs. The excellent biocompatibility of CS can reduce the cytotoxicity of AMPs, enhancing the stability of drug loading and simultaneously improving the therapeutic effect [127]. Piras et al. explored whether the release of AMPs is controllable by CS-NPs, and the result was that the AMPs were stably and linearly released [128]. Piras also loaded frog skin-derived AMP TP into CS-NPs, identifying higher antimicrobial activity and lower toxicity [129]. It is clear that AMP loaded by CS shows broad clinical application prospects.

## 5. Advantages and Disadvantages

According to various studies CS, and its derivatives have been identified as excellent biomaterials with broad application prospects in the fields of antibacterial and infection control [130]. First of all, CS comes from a vast range of sources and easy to prepare. As a natural polymer extracted from chitin and produced by the deacetylation process, it is biodegradable, antigen-free, non-toxic, and has good biocompatibility, as well as significant natural antibacterial properties. When its unsatisfying solubility in water and common organic solvents restrain its use, findings show that three of its nucleophilic functional groups allow it to be easily grafted and modified, so as to obtain various abilities [42]. Therefore, on the premise of retaining the antibacterial property, chitosan derivatives can not only improve the shortcomings of CS, but also receive more biological properties, such as prolonged drug release time [33], through the introduction of modification groups, making its range of application even wider.

Secondly, CS and its derivatives have broad-spectrum antibacterial properties, and it is hard for bacteria to develop drug resistance when the drug exerts antibacterial effects. It has been reported that CS and its derivatives display direct antibacterial activity against an abundance of microorganisms, including Gram-positive and Gram-negative bacteria, reflecting their strong broad-spectrum antibacterial activity, although differences do exists between their structures [42]. In addition, owing to the unique mechanism of CS and its derivatives regarding their antibacterial properties, i.e., the unique cationic characteristics of CS and its derivatives attract and interact with the negatively charged bacteria through electrostatic interaction, they can enter the bacteria and bind to DNA and can also form a barrier outside the bacteria to prevent bacteria from taking up nutrients [33]. Under this mechanism, bacteria can maintain their sensitivity to these antibacterial agents without developing drug resistance [62].

CS and its derivatives also exhibit significant advantages as carriers in antimicrobial therapy, whereas excellent biocompatibility is one of its highlights. At the same time, their mucosal adhesion, permeability enhancement, and controlled drug release through ion interaction make them stand out among drug delivery systems [21]. The primary amino groups of CS allow it to attach to small-molecule drugs [41], so CS and its derivatives can deliver drugs in various forms, such as through microsphere or nanoparticle encapsulation, gel, film, etc. [98]. As for their controlled drug release property, they can be modified by cross-linking with different molecules, thanks to their amine and hydroxyl functional groups. In this way, higher mechanical strength and a more controlled drug release rate will be achieved [131].

In spite of their excellent performance, there are still shortcomings and problems to be solved in the research progress regarding CS and its derivatives, such as the possible impact on the environment during chitin extraction and deacetylation, the higher production costs compared with those of other polymers, the solubility at physiological pH, etc. [27,132,133]. Although there have been many studies on the antibacterial properties of CS and its derivatives, few have been applied to clinical research. The potential immunogenicity, long-term toxicity, and target specificity of CS and its derivatives still need to be improved [134]. The advantages and disadvantages are summarized in Table 1.

## 6. Conclusions and Perspectives

Chitosan, a natural polymer, has attracted significant attention for its distinctive cationic properties and favorable biological characteristics. Numerous studies have revealed a range of advantages of CS, including potent broad-spectrum antibacterial properties, biodegradability, and biocompatibility. Despite its limited solubility as a drawback, this can be easily modified by introducing functional groups into CS, which has also achieved new functionalized derivatives that exhibit new mechanisms for direct antibacterial activity. Furthermore, it has been found that the antibacterial effects of diverse types of CS and its derivatives vary among different bacteria. Their excellent biocompatibility makes them suitable as drug carriers in antibacterial therapy, helping to enhance their therapeutic efficacy while reducing drug toxicity. In this regard, understanding the synergistic effect of chitosan with antibiotics and the effect of chitosan particles on antibacterial efficacy may be of great interest in the future. Although current research on CS and its derivatives still stagnates in experimental stage and lacks clinical applications, with their potential immunogenicity and long-term toxicity risks under exploration as well, the ease of modification of CS allows for improvement in almost all of its properties, which may generate ongoing interest in its further development. We remain optimistic about their future, while it cannot be denied that there is still a long way to go.

## Figures and Tables

**Table 1:** Advantages and disadvantages of CS and its derivatives.

Advantages of CS and its derivatives	Easily sourced Easy to prepare
	Good biodegradability and biocompatibility
	Strong natural antibacterial properties Broad antibacterial spectrum Not suseptsable to causing bacterial resistance
	Can be modified to facilitate the introduction of functional groups Can be grafted with metals and inorganics to change its properties
	Good mucosal adhesion and permeability enhancement
	Controlled drug release
	Multisite loading of drugs
Disadvantages of CS and its derivatives	Low solubility at physiological pH
	Preparation process may cause environmental contamination
	Lack of clinical study results
	Lack of clinical chitosan-based drugs
	Some derivatives may be toxic
	Long-term toxicity in organisms is unknown
	Less suitable for systemic infections Effect is often localized
